# Advancing early onset colorectal cancer research: research advocacy, health disparities, and scientific imperatives

**DOI:** 10.3389/fonc.2024.1394046

**Published:** 2024-07-19

**Authors:** Andrea J. Dwyer, Aniruddha Rathod, Carli King, F. E. R. Vuik, Phuong Gallagher, Anjee Davis, Eric M. Lander, Jose Perea

**Affiliations:** ^1^ Community and Behavioral Health, University of Colorado, Denver, CO, United States; ^2^ Peter O’Donnell Jr. School of Public Health, UT Southwestern Medical Center, Dallas, TX, United States; ^3^ Research Advocacy, Fight Colorectal Cancer, Springfield, MO, United States; ^4^ Department of Gastroenterology and Hepatology, Erasmus Medical Center, Rotterdam, Netherlands; ^5^ Minnesota Oncology Hematology PA, Minneapolis, MN, United States; ^6^ Institute of Biomedical Research, University of Salamanca, Salamanca, Spain

**Keywords:** early age onset colorectal cancer, EAOCRC, EOCRC, young colorectal cancer, colorectal cancer, research early onset colorectal cancer

## Abstract

Early onset colorectal cancer (EOCRC) emerged as the fourth foremost contributor to cancer-related mortality among both genders in the late 1990s. Presently, EOCRC (<50) ranks as the leading cause of cancer mortality in men and the second leading cause in women within the United States. Similar trends are now also evident globally, particularly in developed countries. Furthermore, there is strong evidence confirming that health disparities persist in the diagnosis and treatment of EOCRC, with signs indicating that these gaps may worsen in specific cases. These alarming trends highlight the critical need for research to inform evidence-based interventions to reduce the burden of EOCRC globally. Fight Colorectal Cancer (Fight CRC) is the leading patient advocacy group in the United States providing information on colon and rectal cancer research, prevention, treatment, and policy. It is the opinion of Fight CRC that an international, coordinated effort with the medical, research, scientific, advocacy, industry and funding community is needed to advance impactful research. Fight CRC, in partnership with José Perea, MD, PhD, of the Institute of Biomedical Research of Salamanca (IBSAL) in Spain, and partners, are working together to address this global phenomenon and are presenting a multi-faceted research approach to move the field forward.

## Introduction

Fight CRC hosted a research convening, entitled, “the Early Age Onset Think Tank” on December 1, 2023, in collaboration with the National Cancer Institute (NCI) and Vanderbilt University, assembling nearly 60 scientists, clinicians, and advocacy and policy experts to discuss shared strategies to further the research on the etiology of EOCRC and to examine the interventions that can reduce incidence and mortality (1). The December 2023 meeting expanded upon the outcomes and themes from the foundational Fight CRC workgroup meeting in February 2019 (2). This earlier meeting sparked a call to action within the research community, prompting Fight CRC to regularly convene an EOCRC working group with José Perea, MD, PhD, the NCI, and various professionals, to advance the research agenda. Understanding the etiology of EOCRC, educating medical providers about EOCRC, and establishing international cohorts were amongst the high priority action items discussed (3). Examples of key events and activities that have helped progress the initiatives or brought heightened awareness to EOCRC within the last three years are highlighted in **Table 1**.

**Table 1:** Fight colorectal cancer early onset key events, activities and progress.

Initiative	Examples of Key Events, Activities and Progress
Understanding the etiology of EOCRC.	• Peer-reviewed publications and publicly available data sets • Fight CRC and NCI Think Tanks and symposiums (1) • Research funding and Notice of Special Interest (NOSI)
Educating medical providers about EOCRC.	• High profile deaths of EOCRC patients, such as Chadwick Boseman, bringing awareness to EOCRC, particularly within the African American community • Data releases such as the American Cancer Society’s “Facts and Figures” with routine updates on incidence and mortality (4) • Guideline changes and education campaigns for colorectal cancer screening from 50 to 45 years of age, based on the American Cancer Society (5) and United States Preventive Services Task Force (6) recommendations
Establishing international cohorts.	• Fight CRC Think Tanks and international partnerships with José Perea, MD, PhD, and research and advocacy communities • International research funding

To build on this foundational work and continue to propel the research agenda, Fight CRC conducted virtual, nominal group technique (NGT) sessions in 2023 to reexamine the key areas for resource allocation and research priorities. The NGT, as described by the Centers for Disease Control and Prevention, is an efficient method used to prioritize topics and to have weighted feedback from the group (7). In total, 36 experts participated in the NGT process. There was a diverse mix of medical and scientific professionals, with about one-half of the group comprising medical gastroenterologists, epidemiologists, and oncologists, and a smaller representation of primary care physicians, microbiologists, and biostatisticians. In addition, trained research patient advocates also participated in the process.

Two key areas emerged as priorities for further discussion and were the dedicated area of discussion:

1. EOCRC etiology and the role of exposures
2. Targeted interventions to address the increasing incidence and mortality of EOCRC

The December 2023 Early-Age Onset Think Tank agenda was developed based on this feedback and included two tracks to discuss the research opportunities available within the areas noted above. Presentations from experts on current funding opportunities were provided and given to build the framework required for discussions on the research opportunities. There were dedicated note takers who captured the key themes of each speaker, the questions addressed and the overarching themes of each track. The research themes and priorities noted herein were based on the key considerations and focal points noted in each session and throughout the meeting.

## Key takeaways from the Early-Age Onset Think Tank

Each track was enriched with expert insights and the introduction of novel scientific approaches and technologies, while maintaining relevance to patients with a research advocate. Some of these strategies have been applied in other cancers but are now being considered for CRC, particularly in understanding etiology and developing interventions for EOCRC. Discussions also emphasized inclusivity, with a focus on ensuring equity and access in research and interventions, addressing disparities, and enhancing engagement across diverse populations. Patients who have personally experienced EOCRC and have become trained research advocates through Fight CRC’s Research Advocacy Training & Support (RATS) program contributed insights and perspectives during discussion. This inclusion aimed to ground the scientific discussions in real-world experiences, addressing prevalent concerns and themes that emerged during the sessions.

### Current funding opportunities

There are opportunities for international funding that are open to investigators worldwide, while other international funds require collaboration with a specific country or countries. In the United States, the National Institutes of Health (NIH), the NCI, the Department of Defense’s Congressionally Directed Medical Research Program (DoD CDMRP), and the National Institute of Environmental Health Sciences (NIEHS) all offer grants spanning the entire continuum of EOCRC research, including funds to facilitate meetings and network expansion. The need for collaboration and innovative new approaches to EOCRC research with the inclusion of trained research advocates were continuing themes.

### The state of the Science: EOCRC

A significant rise in colorectal cancer cases has been observed among younger individuals, with cases in those under 55 years increasing from 11% in 1995 to 20%, and cases under 65 years from 27% to 45% (4). These trends are now also evident globally, particularly in developed countries (8). Screening uptake remains critically low, with only 2% of the population aged 45–49 undergoing stool testing in 2021, pointing to a vital area for improvement in early detection efforts (9). A notable observation is the increase in left-sided tumors, especially in individuals aged 45–49, where the majority of EOCRC cases are found, suggesting a potential area for targeted research and intervention (10, 11). The data also highlights significant disparities in EOCRC disease burden, with Native Americans experiencing the highest incidence and Black individuals having the lowest survival rates (4, 12). Additionally, there are significant racial disparities in treatment, underscoring the need for equity in healthcare access and treatment strategies (13). According to Fight CRC’s polling of the patient and survivor community prior to the meeting, 80% of the 350 responding patients indicated that they had to see two or more doctors before getting the appropriate screening and diagnosis (14)

### Research exploration and health disparity considerations

A retrospective study suggested that a striking 50% of EOCRC could have been diagnosed earlier and roughly 15% potentially prevented if current guidelines for colorectal cancer screening had been followed (15). Given that individuals with EOCRC are often diagnosed at advanced disease stages, this study highlights the importance of timely colorectal screening among young adults and those at high risk (10). There remains an unmet need to effectively implement risk-based stratification and population-based intervention strategies to improve adherence to current guidelines and mitigate the alarming increase in EOCRC incidence and mortality. EOCRC also has a multifactorial etiologic mechanism and understanding these etiologies involving environmental exposures, metabolomics, microbial factors and epigenetics is important for preventive and therapeutic purposes. Additionally, at least 75% of EOCRC cases are not heritable, and therefore may be related to the interplay between the genome and environmental exposures that accrue throughout life (16, 17). The emerging field of exposome oncology in EOCRC offers the potential to identify suspect exposures and link exposures with mechanisms of carcinogenesis to ultimately discover modifiable risk factors for EOCRC.

Below are key areas that were the primary key areas for research interventions and underlying health disparities that were discussed at the meeting and in the literature that must be considered as we move toward risk-based stratification and study of etiology (**Table 2**).

**Table 2:** Targeted early onset colorectal cancer research and interventions.

Research and Intervention Focus	Description	Health Disparity Considerations For Further Research
Population adherence to screening guidelines	In the United States, CRC screening is recommended to begin at the age of 45 years for individuals at average risk. Screening in eligible young adults remains low.	• Adherence to CRC screening recommendations vary significantly by age, race/ethnicity, immigration status, education, insurance status, and geography (11). • Young adults aged 45–49 years (20%) or 50–54 years (50%) have lower screening uptake relative to adults aged 55 years and older (9, 11). • Amongst eligible young adults aged 45–49 years, screening was lowest in uninsured individuals (7.6%), individuals with less than a high school diploma (15.4%), and Asian individuals (13.1%) (9). • Asian (50%), Hispanic (52%), and American Indian/Alaska Native (52%) individuals that are eligible for screening (≥45 years) have lower screening prevalence relative to White (61%) or Black (61%) individuals (11). • Of those individuals eligible for screening (≥45 years), uninsured individuals (21%), individuals residing in the United States less than 10 years (29%), and individuals with less than a high school education (48%) also have low CRC screening uptake (11). • In the United States, screening rates in those eligible for screening (≥45 years) vary drastically by state (from 53% in California to 70% in Washington DC and Massachusetts)^12.^ • Primary screening modality, age of initiation, and guideline recommendations range by country. Adherence to CRC screening guidelines varies by country (from 19% in Croatia and the Czech Republic to 69% in the Basque region of Spain) (18).
Utilization of emerging screening modalities	Emerging technologies for stool- and blood-based screening methods are promising. However, stool-based CRC screening methods are under-utilized in young adults.	• Utilization of stool-based tests are significantly lower in individuals aged 45–49 years (3%) compared to older adults (9 to 15%) (11). • African American and Asian individuals have lower rates of a follow-up colonoscopy after a positive stool-based test relative to White individuals (19). • Follow-up colonoscopy rates are associated with the insurance type, health care organization, and type of stool-based test utilized (19).
Understanding family history of CRC	Earlier screening and more intensive surveillance are recommended for individuals with a family history of CRC and advanced adenomas (20). However, untimely screening in high-risk individuals remains a concern.	• Family history of CRC is more likely to be underreported by minority populations (21). • Significant disparities exist in the availability and completeness of cancer family history information within electronic health records by sex, race and ethnicity, and language preference (22). • African Americans and Hispanics are less likely to know their family history of CRC and are less likely to undergo screening despite a known family history (23).
Determining the etiologic mechanisms of EOCRC	There are multiple mechanisms that contribute to EOCRC pathogenesis. The interplay between contributing factors – environmental, metabolic, microbial, and genetic – warrants further investigation.	• EOCRC studies utilizing tumor phenotyping in minority groups is lacking, both domestically and internationally (23). • African Americans and Hispanic individuals are less likely to be referred for genetic testing (23). • Exposure to risk factors varies significantly by race and ethnicity (23). • African Americas and Hispanic populations have a higher prevalence of childhood obesity and young-onset type II diabetes (23).

#### Population adherence to current screening guidelines

Adherence to CRC screening guidelines in eligible adults at average-risk (≥45 years) varies by age, race and ethnicity, geography, and socioeconomic status (11). Screening in eligible young adults remains low, with only 20% of adults aged 45 to 49 years and 50% of adults aged 50 to 54% being up to date on CRC screening in 2021 (11). Additional racial, ethnic, and socioeconomic disparities within this young adult population with already low uptake of CRC screening increases health inequities (9).

#### Utilization of emerging screening modalities

The emergence of new screening technologies and the option for multiple screening methods is promising for increasing screening uptake. However, the uptake of current stool-based screening methods is low (3%) in eligible adults aged 45 to 49 years compared to older adults (11). Notable racial disparities exist in the rate of follow-up colonoscopy within one year of a positive result using a stool-based test (19). As additional screening modalities are introduced, it must be recognized that these methods will not alone resolve the existing disparities in screening uptake and follow-up adherence.

#### Understanding family history of CRC

Family history is an established risk factor for EOCRC and guidelines exist for earlier screening and more intensive surveillance in individuals with a family history of CRC or advanced adenomas (20). The likelihood to know and report family history, as well as to undergo screening despite a known family history, varies by race and ethnicity (21, 23). These disparities likely contribute to the varying availability and completeness of cancer family information within electronic health records, which have been observed to vary by race, ethnicity, and language preference (22).

#### Determining the etiologic mechanisms of EOCRC

Multifaceted causality in EOCRC is not yet established, in part due to absence of longitudinal multiomics data antecedent to CRC. Efforts which map out the development of colorectal cancer are emerging, and it is known that the majority of CRC (all populations) share similar deleterious somatic mutations (24, 25). Future studies are required to unravel the intricate interplay among the exposome, microbiome, epigenome, proteome, and transcriptome to understand what may be hastening epigenetic drift and promoting acquisition of mutations in known CRC driver genes. The discovery of these oncogenic mechanisms will direct development of targeted screening modalities, therapeutic treatments and risk prediction with the vital objective of enhancing patient care and outcomes.

## Discussion

A key insight from the discussions is the imperative to integrate scientific disciplines from both scientific tracks to enhance our understanding of interventions and risk-based stratification, necessitating a deeper knowledge of the exposome and biological factors. This interdisciplinary approach is crucial for advancing risk stratification methods and will be a focus of upcoming convenings. Underscoring the need for further examination and research in health disparities will be a primary focus.

Significant targeted measures, such as those focused on reducing disparities and increasing adherence, are required to reverse the striking increase in EOCRC incidence and mortality. We have identified key areas for research areas in which these targeted measures have the potential to reduce the existing health disparities and inequities, highlighted in **Table 2**. Strategies to improve adherence to screening guidelines amongst the eligible average-risk population include organized screening programs, patient navigators, media campaigns, patient reminders, financial incentives, and multiple options of screening methods offered to eligible patients. However, there is a need to identify the barriers associated with low screening prevalence in the young adult population so that recommendations may be tailored to this population. With the emergence of new screening methods, substantial systemic efforts are required to address low uptake of these tests among young adults and disparities in follow-up care.

As we move towards risk-stratified screening strategies, further research is required to understand the racial, ethnic, geographical, and socioeconomic disparities in exposure to and care associated with risk factors. Inadequate inquiries about family history of CRC or advanced adenomas and age of cancer onset likely contributes to untimely screening for increased-risk individuals. One study reported only 70% of primary care physicians routinely recommended CRC screening in young adults (40-to-49 years of age) with a first degree relative with colorectal cancer (26). Future studies assessing the barriers limiting inquiries (i.e. time with patients, unknown family history, lack of knowledge on screening guidelines, etc.) are necessary to inform recommendations for intervention. Systemic efforts are required to address the racial and ethnic inequities associated with knowing, reporting, and documenting family history of CRC. The development and implementation of a standardized system, such as a national CRC registry or integration within the electronic health record management systems, that allows for the standardized collection of family history and communication of increased risk to individuals with a family history is critical.

Determining the etiologic mechanisms of EOCRC is extremely complex and will require the collaboration of interdisciplinary and interagency collaborations to consider industry, medical, environment and advocacy community to strongly collaborate internationally. In addition to the specific research strategies to examine a multifaceted causality, there will be the need for infrastructure support for the creation of standardization of epidemiologic research instruments, well-resourced biorepositories with allowance for strong access and transfer of data and specimens for the strongest research approaches to adequately research (2).

As a patient advocacy community, Fight CRC feels it is imperative to infuse the patient voice in the research discovery phase, as well as the design and in research implementation. Fight CRC embraces the engagement of the training of research advocates and integrating the scientific and advocacy community to infuse the voice of the patient communty (27). It is the opinion of Fight CRC, that advocacy partners, funders, patients, and the scientific community must unite to achieve worldwide focus and propel the quest to mitigate and ultimately eradicate colorectal cancer through shared knowledge and collaborative scientific efforts.

Moving forward, Fight CRC with José Perea, MD, PhD, and collaborators plan to extend our research advocacy efforts through a global Early-Age Onset Think Tank series leading to an intensive session in person in Europe of June 2025. This initiative aims to further prioritize and define the research agenda by uniting experts across the oncology spectrum to sustain dialogue and innovation to continue the convening power established over the last five years.

## Data availability statement

The original contributions presented in the study are included in the article/supplementary material. Further inquiries can be directed to the corresponding author.

## Author contributions

AJD: Conceptualization, Data curation, Formal analysis, Investigation, Methodology, Project administration, Resources, Software, Supervision, Validation, Visualization, Writing – original draft, Writing – review & editing. AR: Conceptualization, Data curation, Investigation, Writing – original draft, Writing – review & editing. CK: Conceptualization, Data curation, Formal analysis, Methodology, Project administration, Writing – original draft, Writing – review & editing. FV: Data curation, Writing – original draft, Writing – review & editing. PG: Writing – original draft, Writing – review & editing. AD: Conceptualization, Writing – original draft, Writing – review & editing. EL: Conceptualization, Methodology, Writing – original draft, Writing – review & editing. JP: Conceptualization, Investigation, Validation, Writing – original draft, Writing – review & editing.
